# Study on the Influence Mechanism of the Rhizosphere Soil Microbial Community and Physicochemical Factors on the Occurrence of Pepper *Phytophthora* Blight

**DOI:** 10.3390/microorganisms13122765

**Published:** 2025-12-04

**Authors:** Xin Wang, Fan Yang, Ying Zhang, Miaomiao Liu, Yuting Hong, Xiaoke Chang, Hongxun Jiang, Wenrui Yang, Qiuju Yao, Baoming Tian

**Affiliations:** 1 Institute of Vegetable, Henan Academy of Agricultural Sciences, Graduate T&R Base of Zhengzhou University, Zhengzhou 450002, China; wx269152874@163.com (X.W.); xiaoyuefuxiang@163.com (F.Y.); nkyzhy@126.com (Y.Z.); liumm2023@163.com (M.L.); hongyt182808@163.com (Y.H.); cxk8802@163.com (X.C.); jianghx1712@163.com (H.J.); yangwenrui1118@163.com (W.Y.); 2 School of Agricultural Sciences, Zhengzhou University, Zhengzhou 450001, China

**Keywords:** pepper, *Phytophthora capsici*, high-throughput sequencing, rhizosphere microorganisms, soil enzyme activity

## Abstract

In order to clarify the changes and correlations among microbial community structure and soil environmental factors in the rhizosphere soil of peppers under healthy and diseased conditions, Illumina MiSeq technology was used to perform high-throughput sequencing of the V3-V4 region of the bacterial 16S rRNA gene and the ITS hypervariable region of fungi in the rhizosphere soil of peppers. The dominant species and key environmental factors affecting the occurrence of pepper *Phytophthora* blight were analyzed and screened, and the functions of bacteria and fungi in the samples were predicted by PICRUSt2 and FUNGuild. The results showed that except for soil pH, the contents of microbial biomass carbon, magnesium, zinc, and iron in the rhizosphere soil of healthy peppers were significantly higher than those in the diseased soil. Alpha diversity analysis showed that the diversity index of the bacterial community in healthy soil was higher than that in diseased soil, while the diversity index of the fungal community was significantly lower than that in diseased soil. The relative abundance of beneficial bacteria such as Proteobacteria, Actinobacteriota, Burkholderiales, and Rhodanobacteraceae in the rhizosphere soil of healthy peppers was higher. Pathogens such as *Penicillium* and *Fusarium* were significantly enriched in the rhizosphere soil of diseased pepper plants. The functional prediction results showed that soil bacteria were mainly metabolized, including the biosynthesis of ansamycin, the biosynthesis of vancomycin antibiotics, the biosynthesis of valine, leucine, and isoleucine, the metabolism of C5-branched dicarboxylic acid, and the biosynthesis of fatty acids. The main nutritional strategies of the fungal community are disease prototype and saprophytic. Combined with the key environmental factors, microbial composition, and correlation analysis of pepper rhizosphere soil, it is speculated that the occurrence of pepper *Phytophthora* blight may be related to the synergistic effect of soil nutrients and microbial flora, which provides a theoretical basis for the biological control of pepper *Phytophthora* blight in the future.

## 1. Introduction

Peppers (*Capsicum annuum* L.) are an important annual or perennial herb, which are often rich in vitamin C, vitamin E, β-carotene, lutein, capsaicin, flavonoids, and other mineral nutrients and bioactive components [1]. In recent years, in the process of pepper cultivation, excessive fertilization, long-term continuous cropping, and intensive planting have led to soil nutrient imbalance, decreased organic matter content, and increased accumulation of soil-borne pathogens, which has seriously hindered the high-quality production of peppers [2]. Pepper *phytophthora* blight, also known as black rod disease, is an oomycete disease caused by *Phytophthora capsici* Leonian. It has the characteristics of strong pathogenicity, fast propagation speed, large damage range, and difficult control [3]. The disease is prone to high incidence under hot (25–28 °C) and humid conditions, mainly in the stems, fruits, leaves, and other parts of peppers, often causing plant wilting and death, and can lead to total yield loss of peppers in severe cases [4].

At present, conventional methods such as pesticide application, grafting, and rotation can prevent and control pepper *Phytophthora* blight to a certain extent, but in practical applications these methods may lead to an increase in production costs. The long-term use of chemical pesticides may also induce pathogen resistance, which leads to a vicious cycle of increasing dosage and increasing environmental pollution [5]. Biological control is an efficient, green, and environmentally friendly prevention and control technology, which can effectively utilize beneficial microorganisms such as *Bacillus* [6], *Trichoderma* [7], *Actinomycetes*, etc. [8]. These bacteria can produce metabolites such as enzymes and antimicrobial peptides to inhibit the growth of pathogens [9].

As a key area of root–soil–microorganism interaction, the rhizosphere is not only the most intensive area of material exchange between plants and soil, but also one of the most complex ecosystems in the world [10]. Changes in the physical and chemical properties of rhizosphere soil usually have a significant effect on plant health and soil microbial diversity [11]. Liu et al. suggest that changes in soil pH, nitrogen, and salt content will lead to changes in the microbial community structure and related species composition in the rhizosphere soil of American ginseng, which will affect its health [12]. Rhizosphere microorganisms play an important role in regulating plant growth and increasing soil nutrient utilization. These microorganisms can promote plant nutrient absorption through specific metabolic activities and produce antagonistic substances to inhibit soil-borne pathogens and systematically enhance host disease resistance [13]. For example, the inoculation of functional strains in the rhizosphere of peppers can directionally regulate microbial community structure, improve soil fertility, and effectively resist pathogen infection [14]. The application of microbial agents can also reshape the tomato rhizosphere microbiome, significantly reduce the incidence of disease, and increase yield [15]. The application of *Rhodopseudomonas palustris* PSB06 can not only improve the richness of microbial community, but also specifically increase the abundance of actinomycetes so as to realize the biological control of pepper blight [16]. Although the above studies have confirmed the disease prevention potential of rhizosphere microorganisms, current research on peppers, both domestically and internationally, primarily focuses on disease prevention efficiency and aboveground traits or microbial community analysis based on traditional culture methods [17]. Moreover, research on the microbiome of pepper *Phytophthora* blight is still limited. Therefore, through high-throughput sequencing technology, the bacterial community in the rhizosphere of peppers can be comprehensively identified and analyzed, and the differences in the microbial community structure in the rhizosphere soil between the diseased and healthy areas of pepper *Phytophthora* blight can be explored, so as to determine the key differential groups. Furthermore, the key factors affecting the occurrence of pepper *Phytophthora* blight are more accurately uncovered.

This study aims to investigate the differences in rhizosphere microbial community composition between pepper *Phytophthora* blight-affected and healthy regions by combining Illumina second-generation high-throughput sequencing technology with soil physicochemical properties analysis. It seeks to elucidate the interrelationship between rhizosphere soil physicochemical properties, microbial diversity, and pepper *Phytophthora* blight incidence, thereby identifying key environmental factors influencing disease occurrence, uncovering critical microbial communities, advancing green control strategies for pepper diseases, and providing theoretical support for microbial agent-based management of pepper *Phytophthora* blight.

## 2. Materials and Methods

### 2.1. Collection of Rhizosphere Soil

In August 2024, rhizosphere soil samples of peppers were collected at the experimental base of Henan Academy of Agricultural Sciences in Yuanyang County, Xinxiang City, Henan Province (average annual temperature of 14.4 °C, annual rainfall of 645 mm). Two treatments were set up: diseased (100% incidence of pepper *Phytophthora* blight) and healthy (0% incidence of pepper *Phytophthora* blight). Six biological replicates were set up for each treatment. The S-type five-point sampling method was used for each repeated sample [18]: five plants were randomly selected and their 0–4 mm rhizosphere soil (the attached soil was collected with a sterile brush after shaking the roots) was mixed into one sample. The diseased soil samples were numbered YS1–YS6, and the healthy soil samples were numbered YR1–YR6. All samples were placed on ice and transported back to the laboratory and divided into two parts: one part was frozen at −80 °C for DNA extraction, and the other part was dried for soil physical and chemical properties and enzyme activity analysis. The sampling plots were planted with the same variety of pepper all year round, the fertilization management was consistent, and the soil properties and environmental conditions were similar.

### 2.2. Determination of Rhizosphere Soil Physicochemical Properties

In this study, a 10 g soil sample was collected from YR and YS, respectively, to determine 16 soil physicochemical properties and enzyme activities. These indicators include alkali-hydrolysable nitrogen (AN), available phosphorus (AP), available potassium (AK), organic matter (OM), pH, electrical conductivity (EC) value, soil microbial biomass carbon (MBC), calcium (Ca), iron (Fe), zinc (Zn), magnesium (Mg), copper (Cu), soil alkaline phosphatase (S-ALP), soil invertase (S-SC), soil urease (S-UR), and soil dehydrogenase (S-DHA). Among them, alkaline hydrolysis nitrogen was determined by the alkaline hydrolysis diffusion method [19]. AP was determined by molybdenum-antimony anti-spectrophotometry [20]. AK was determined by the four-benzene boron colorimetric method [21]. The contents of Fe, Ca, Mg, Cu, and Zn were determined by atomic absorption spectrophotometry [22]. The pH value was measured by a glass electrode acidity meter [23]. EC was measured by a conductivity monitor [24]. OM was determined by potassium dichromate-sulfuric acid colorimetry. MBC was determined by the chloroform fumigation extraction method [25]. S-SC activity was determined by the 3,5-dinitrosalicylic acid colorimetric method. S-UR activity was determined by the indophenol blue colorimetric method [26]. S-ALP activity was determined by the disodium phenyl phosphate colorimetric method [27]. S-DHA activity was determined by TTC spectrophotometry [28].

### 2.3. Sample DNA Extraction, Amplification and Sequencing

The total genomic DNA of the sample was extracted from 1 g of soil using the CretMagTM Power Soil DNA Kit (CretBiotech, Shanghai, China), and the specific steps were used according to the kit’s instructions. The extracted DNA was detected by 1% agarose gel electrophoresis, and the concentration and purity of DNA were determined by Nano Drop 2000 UV–vis spectrophotometer (Thermo Scientific, Wilmington, NC, USA). Primer pairs 341F (5′-CCTAYGGGRBGCASCAG-3′) and 806R (5′-GGACTACHVGGGTWTCTAAT-3′) were used in the V3-V4 region of the bacterial 16S rRNA gene using soil DNA as a template. ITS1F (5′-CTTGGTCATTTAGAGGAAGTAA-3′) and ITS2R (5′-GCTGCGTTCTTCATCGATGC-3′) were amplified by PCR. The PCR reaction system was as follows: 25 μL 2 × ES Taq MasterMix (Dye), 2 μL forward primer (10 μM), 2 μL reverse primer (10 μM), 10 ng template DNA, and finally, dd H_2_O was added up to 50 μL. The PCR expansion program was as follows: Pre-denaturation was carried out at 94 °C for 2 min, followed by 30 cycles each including 30 s of denaturation at 94 °C, 30 s of annealing at 55 °C, and 45 s of extension at 72 °C. Finally, a single extension was performed at 72 °C for 10 min, and the reaction was terminated at 4 °C. The above PCR reaction was repeated three times. PCR products were extracted from 2% agarose gel and purified using AxyPrep DNA Gel Extraction Kit (Axygen Biosciences, Union City, CA, USA) according to the instructions of the kit. Quantus TM fluorescent agent (Promega, Madison, WI, USA) was used for quantitative analysis. The Illumina MiSeq PE300 platform (Illumina, San Diego, CA, USA) was used to sequence qualified libraries. All raw sequencing data have been uploaded to the NCBI sequence reading archive database (accession numbers: PRJNA1333628 and PRJNA1333613).

### 2.4. Analysis of Microbial Diversity of Samples

The original 16S rRNA gene sequencing reads were filtered by fastp (version 0.20.0) [29] and spliced using the FLASH program (version 1.2.7) [30]. UPARSE (version 7.1) [31] was used to cluster OTUs with a similarity of 97% and to identify and remove chimeric sequences. RDP Classifier (version 2.2) [32] was used to analyze the taxonomy of each OTU representative sequence using a confidence threshold of 0.7 for the 16S rRNA database (Silva v138) and the UNITE database (https://unite.ut.ee/, accessed on 8 October 2024). Alpha and beta diversity were calculated using QIIME2 software (version 2024.1). The PICRUSt software (version 2.5.3) package and FUNGuild software (version 1) were used to predict the function of bacteria and fungi in soil samples. R language tools were used to draw dilution curves, species composition histograms, and species function histograms.

### 2.5. Statistical Analysis

Statistical analysis was performed using IBM SPSS software (IBM, V.20.0, New York, NY, USA) and R software (version 3.5.2). The microbial community function was analyzed by one-way analysis of variance (ANOVA) and post hoc multiple comparison tests (Tukey HSD). The independent sample *t*-test was used to evaluate the effects of pepper *Phytophthora* blight on soil physical and chemical properties and enzyme activities, the Shapiro–Wilk test was used to verify normality, and the Levene’s test was used to verify the homogeneity of variance. The microbial α-diversity between groups was determined using the Kruskal–Wallis test. The relative abundance differences between the two groups at the phylum and genus levels were analyzed by the Wilcoxon rank sum test. In this study, all statistical tests were considered to be insignificant when *p* > 0.05; when *p* < 0.05, it is considered significant; and when *p* < 0.01, it is considered to be extremely significant.

## 3. Results

### 3.1. Analysis of Physicochemical Properties and Enzyme Activity of Diseased YS and Healthy YR Rhizosphere Soil

**Figure 1:**
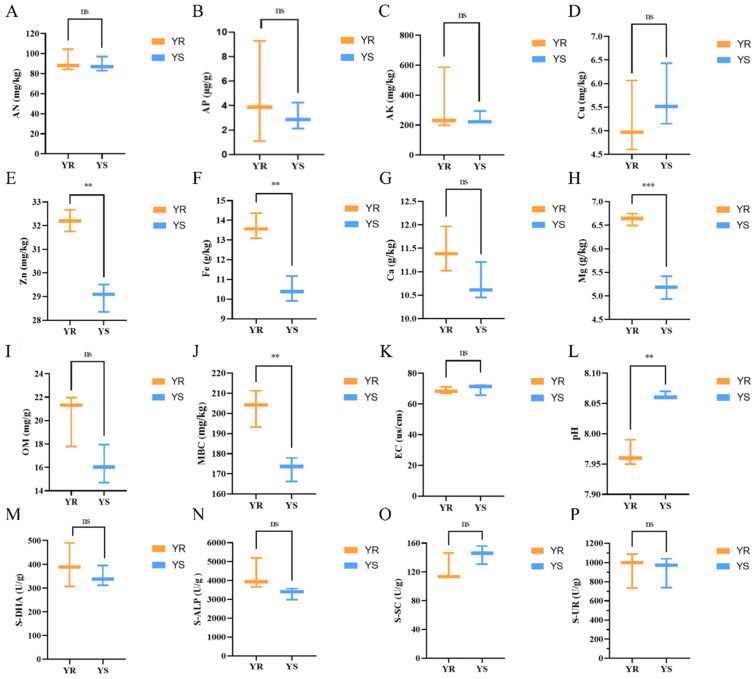
Physicochemical properties and enzyme activities of rhizosphere soil of diseased YS and healthy YR pepper plants. (**A**) Alkali-hydrolysable nitrogen (AN); (**B**) available phosphorus (AP); (**C**) available potassium (AK); (**D**) copper (Cu); (**E**) zinc (Zn); (**F**) iron (Fe); (**G**) calcium (Ca); (**H**) magnesium (Mg); (**I**) organic matter (OM); (**J**) soil microbial biomass carbon (MBC); (**K**) electrical conductivity (EC) value; (**L**) pH; (**M**) soil de-hydrogenase (S-DHA); (**N**) soil alkaline phosphatase (S-ALP); (**O**) soil invertase (S-SC); (**P**) soil urease (S-UR). Significant differences between groups were determined by independent samples *t*-test (*n* = 6, *p* < 0.05). ns, not significant; ** *p* < 0.01, *** *p* < 0.001.

The results of the rhizosphere soil physicochemical properties and enzyme activity of YS in the diseased area and YR in the healthy area are shown in Figure 1. The contents of EC, S-SC, and Cu in YR were lower than those in YS soil, while the contents of AN, S-DHA, AP, S-ALP, OM S-UR, Ca, and AK in YR were higher than those in YS soil, but there was no significant difference (*p* > 0.05). Among the remaining five indicators of soil pH, MBC, Mg, Zn, and Fe, the pH value of YR was 7.967, which was significantly lower than the pH value of YS 8.063 (*p* < 0.05), a decrease of 1.191%. The MBC content of YR was 202.881 mg/kg, which was significantly higher than that of YS (172.578 mg/kg) (*p* < 0.05), which was increased by 17.56%. The contents of Mg, Zn, and Fe also showed the same trend, and YR increased by 27.95%, 11.12%, and 30.30%, respectively, compared with YS.

### 3.2. Microbial Sequencing Data Quality Control and OTU Basic Analysis for Diseased YS and Healthy YR Rhizosphere Soil

**Figure 2:**
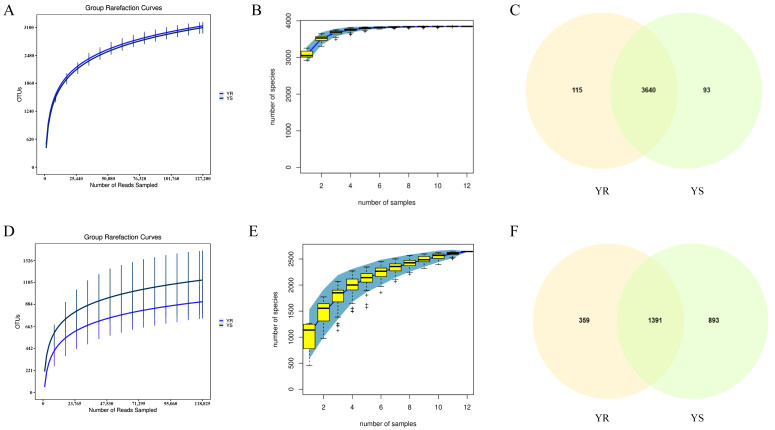
Data quality control and OTU distribution plots. (**A**) Bacterial sparsity curve; (**B**) bacterial species accumulation curve; (**C**) bacterial OTU distribution plot; (**D**) fungal sparsity curve; (**E**) fungal species accumulation curve; (**F**) fungal OTU distribution plot. Note: + represents the box line graph outliers.

Sequencing data from two groups of samples—infected and healthy plants’ rhizosphere soil—underwent quality control. The total number of valid bacterial and fungal sequence reads obtained from all samples was 2,729,875 and 3,824,997, respectively. Among these, the valid sequence reads for bacteria and fungi in infected plants were 1,378,108 and 1,539,257. The bacterial and fungal valid sequence reads for non-infected plants were 1,351,767 and 2,285,740, respectively. Based on the sequencing results, we plotted the sparsity curves and species accumulation curves for bacterial and fungal communities. As shown in Figure 2A,D, the dilution curves for all samples approached a plateau, indicating that the sequencing depth substantially covered all species within the samples. The sequencing results adequately reflected the species diversity present in the current samples, confirming their suitability for subsequent experimental analysis. As shown in Figure 2B,E, when the sample size exceeded 10, the species accumulation curves for both bacteria and fungi tended to flatten, with minimal fluctuations in the number of newly added species. In this study, a sample size of 12 was sufficient to reflect the species composition within the community. As shown by the Venn diagrams in Figure 2C,F, the bacterial community collectively generated 3848 OTUs. Among these, YR contributed 115 OTUs, YS contributed 93 OTUs, and the two shared 3640 OTUs. The fungal community collectively formed 2643 OTUs, with 359 OTUs in YR, 893 in YS, and 1391 shared OTUs between the two.

### 3.3. Analysis of Diversity and Structural Similarity in Bacterial and Fungal Communities of Diseased YS and Healthy YR Rhizosphere Soil

**Figure 3:**
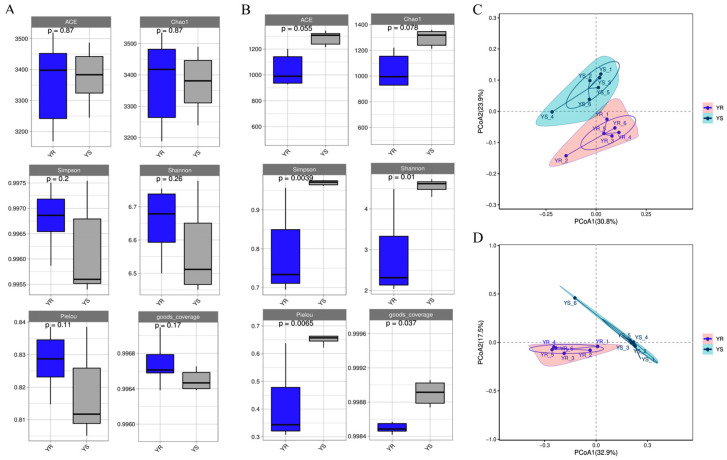
α-diversity and β-diversity of bacterial and fungal communities in YS and YR soils. The box plot shows the changes in the ACE index, Chao1 index, Simpson index, Shannon index, Pielou index and Goods coverage index of bacteria (**A**) and fungi (**B**) in YS and YR. The microbial α-diversity between groups was determined by the Kruskal–Wallis test (*n* = 6, *p* < 0.05). PCoA analysis maps of bacteria (**C**) and fungi (**D**) were drawn based on the Bray–Curtis distance. Among them, the horizontal coordinate represents the first principal component and its contribution to the sample difference; the vertical coordinate represents the second principal component and its contribution to sample differences.

This study employed QIIME2 to calculate six α-diversity indices—ACE, Chao1, Shannon, Simpson, Pielou, and Goods coverage—and plotted box plots to comprehensively assess microbial community richness and diversity. The Goods coverage index indicated that all sample libraries achieved coverage above 0.996, confirming the representativeness of the results for the actual microbial composition in the samples. As shown in Figure 3A, among bacteria, the soil microbiome richness indices (ACE and Chao1) and diversity indices (Shannon and Simpson) in the healthy area were higher than those in the diseased area. However, no significant differences were observed. As shown in Figure 3B, the fungal richness (ACE and Chao1) and diversity (Shannon and Simpson) in healthy regions were lower than in diseased regions. Specifically, the Shannon and Simpson indices indicated that the microbial diversity in YR was significantly lower than in YS (*p* < 0.05), while the Pielou index revealed that the microbial evenness in YR was extremely significantly lower than in YS (*p* < 0.01).

Principal coordinate analysis (PCoA) based on the Bray–Curtis distance was performed on YS and YR samples, with the results shown in Figure 3. For bacteria, the first and second principal coordinate axes explained 30.8% and 23.9% of the variance, respectively (Figure 3C). For fungi, the first and second principal coordinate axes explained 32.9% and 17.5% of the variance (Figure 3D). Regardless of bacterial or fungal groups, intra-group sample points exhibited relative clustering which indicated the high similarity and good reproducibility among samples within each group. Among bacteria, the distribution of sample points between YS and YR showed some separation, suggesting a certain degree of difference in bacterial communities between the two sites, though it was not significant. Among fungi, YS and YR exhibited distinct separation which indicated substantial differences in fungal communities between the two sites. In summary, bacterial and fungal communities in soil samples from diseased areas exhibit distinct differences compared to those from healthy areas. However, environmental factors exert varying influences on bacterial and fungal communities. To clarify the specific effects of healthy versus diseased environments on microbial community composition, further experimental studies are warranted.

### 3.4. Analysis of Species Composition and Differences in Bacterial and Fungal Communities Between Diseased YS and Healthy YR Rhizosphere Soil

**Figure 4:**
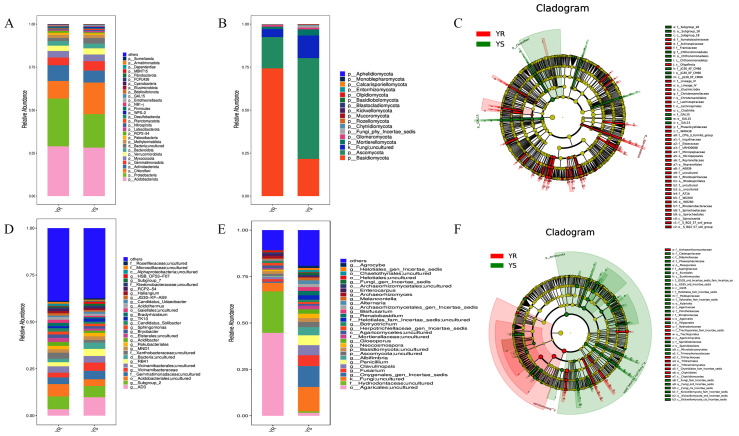
Species composition stacking and Lefse analysis of bacterial and fungal communities in YS and YR soils. (**A**) Distribution map of bacterial phylum-level taxonomic structure. (**B**) Distribution map of fungal phylum-level taxonomic structure. (**C**) Lefse analysis of bacterial communities. (**D**) Distribution map of bacterial genus-level taxonomic structure. (**E**) Distribution map of fungal genus-level taxonomic structure. (**F**) Fungal Lefse analysis. Note: When the *p* value is less than 0.05, the linear discriminant analysis effect value (LEfSe) of bacterial (**C**) and fungal (**F**) communities is greater than 2.0.

To investigate the microbial community composition and differences between the YS and YR regions, we compared the relative abundance of bacteria and fungi at the phylum, order, family, and genus levels (Figure 4). At the phylum level, the bacterial composition with relative abundances exceeding 1% in diseased YS and healthy YR rhizosphere soils is shown in Figure 4A. The five dominant bacterial phyla with the highest relative abundances were as follows: Acidobacteriota, Proteobacteria, Chloroflexi, Actinobacteriota, and Gemmatimonadota. In YR, Acidobacteriota, Proteobacteria, and Actinobacteriota were all higher than in YS, while Chloroflexi and Gemmatimonadota were lower than in YS. At the order level, Vicinamibacterales showed a higher relative abundance in the diseased area, while Acidobacteriales and Rhizobiales were more abundant in the healthy area (Figure S1). At the family level, Gemmatimonadaceae was enriched in the diseased area, while Xanthobacteraceae was enriched in the healthy area (Figure S2). At the genus level, genera with higher relative abundances in YS compared to YR included AD3, Subgroup 2; Acidobacteriales, uncultured; Gemmatimonadaceae, uncultured; and *Vicinamibacteraceae*. Specifically, the genera Subgroup 2 and Acidobacteriales, uncultured, showed higher relative abundances in healthy areas, while the genera AD3; Gemmatimonadaceae, uncultured; and Vicinamibacteraceae exhibited higher relative abundances in diseased areas (Figure 4D). At the phylum level, the fungal composition with relative abundances exceeding 1% in YS and YR rhizosphere soils is shown in Figure 4B. Dominant fungal phyla with higher relative abundances included Basidiomycota, Ascomycota, and Mortierellomycota. Among these, the relative abundance of Basidiomycota was higher in YR than in YS, while Ascomycota and Mortierellomycota were lower in YR than in YS. At the order level, the relative abundances of Agaricales and Trechisporales were higher in the healthy region than in the disease-prone region, while those of Hypocreales and Onygenales were lower in the healthy region (Figure S3). At the family level, healthy areas showed enrichment of Hydnodontaceae, while diseased areas exhibited enrichment of Nectriaceae (Figure S4). At the genus level, dominant fungal genera with higher relative abundances in YS compared to YR primarily included the following: Agaricales, uncultured; Hydnodontaceae, uncultured; *Fusarium*; *Clavulinopsis*; and *Penicillium*. Specifically, Agaricales, uncultured, and Hydnodontaceae, uncultured, were higher in YR than in YS. Meanwhile, the relative abundances of the pathogenic genera *Fusarium* and *Penicillium* were 1.32% and 0.12% in YR, significantly lower than the 5.85% and 5.33% observed in YS (Figure 4E). These results indicate that YS and YR share similar microbial compositions at the phylum and genus levels for dominant bacteria and fungi, but exhibit distinct differences in relative abundance.

Linear discriminant analysis effect size (LEfSe) was used to identify microbial groups that were significantly different between YS and YR (*p* < 0.05), which were designated as unique biological markers (Figure 4C,F). The results indicate that the key bacterial groups in the infected and healthy regions include Chloroflexi, GAL15 (from phylum to order), Micropepsaceae, Micropepsales, and Clostridia. Dominant fungal communities comprised Basidiomycota, Agaricomycetes, Agaricales, Ascomycota, Eurotiomycetes, and Eurotiales. Based on these findings, we infer that the dominant species in the rhizosphere soil of healthy pepper plants are bacteria belonging to the phyla Proteobacteria, Actinobacteriota, Burkholderiales, and Rhodanobacteraceae. Among fungi, the dominant groups are Basidiomycota, Agaricales, and Hydnodontaceae. In contrast, the rhizosphere soil of diseased pepper plants shows enrichment of bacteria belonging to Chloroflexi, *Vicinamibacteraceae*, and GAL15, as well as the fungal Ascomycota, *Penicillium*, and *Fusarium*.

### 3.5. Functional Prediction of Bacterial and Fungal Communities in Diseased YS and Healthy YR Rhizosphere Soil

**Figure 5:**
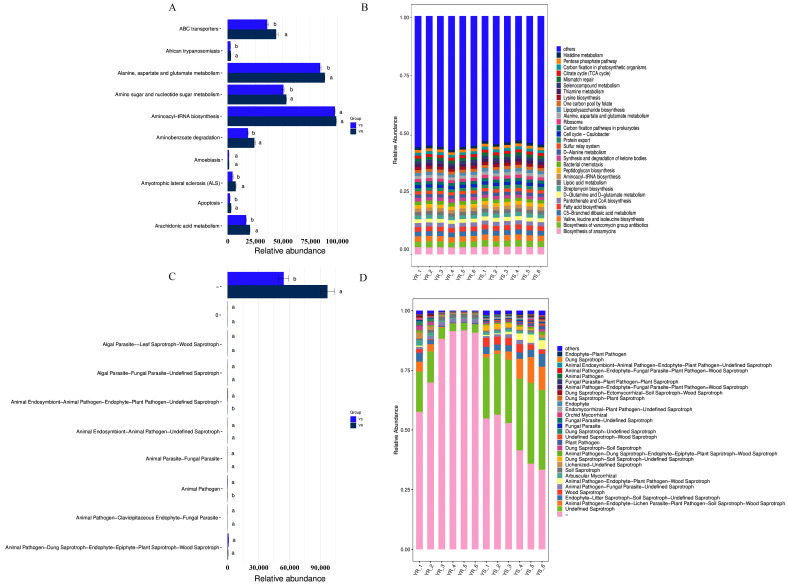
Functional analysis of bacterial and fungal communities in the rhizosphere soil of YS and YR. (**A**) ANOVA analysis of bacterial communities based on functional gene abundance. (**B**) Stacked bar chart of bacterial community functional abundance. (**C**) ANOVA analysis of fungal communities based on functional gene abundance. (**D**) Stacked bar chart of fungal community functional abundance. Note: For (**A**) and (**C**), differences between groups were evaluated using one-way ANOVA followed by Tukey’s HSD test (n = 6, *p* < 0.05). Different letters above bars indicate significant differences.

In order to further analyze the functional differences of soil microbial communities in healthy and infected rhizosphere soil of peppers, PICRUSt2 and FUNGuild were used to predict the functions of the bacterial and fungal communities, respectively. As shown in Figure 5B, the bacterial community exhibited higher functional abundance in the following pathways: biosynthesis of ansamycins and biosynthesis of vancomycin group antibiotics, valine, leucine, and isoleucine biosynthesis, C5-branched dibasic acid metabolism, and fatty acid biosynthesis. Among these, the YS group exhibited relatively higher relative abundances for the biosynthesis of ansamycins and the biosynthesis of vancomycin group antibiotics. Based on ANOVA analysis, we observed that the functional abundance of alanine, aspartate, and glutamate metabolism in the YR group was significantly higher than that in the YS group (Figure 5A).

As shown in Figure 5D, the fungal communities with higher predicted functional abundance primarily included the following: undefined saprotroph; animal pathogen–endophyte–lichen parasite–plant pathogen–soil saprotroph–wood saprotroph; endophyte–litter saprotroph–soil saprotroph–undefined saprotroph; wood saprotroph; and animal pathogen–fungal parasite–undefined saprotroph. Among these, the functional abundance of animal endosymbiont–animal pathogen–endophyte–plant pathogen–undefined saprotroph was significantly higher in YS than in YR (Figure 5C).

### 3.6. Correlation Analysis of Microbial Community Structure with Environmental Factors in Diseased YS and Healthy YR Rhizosphere Soil

**Figure 6:**
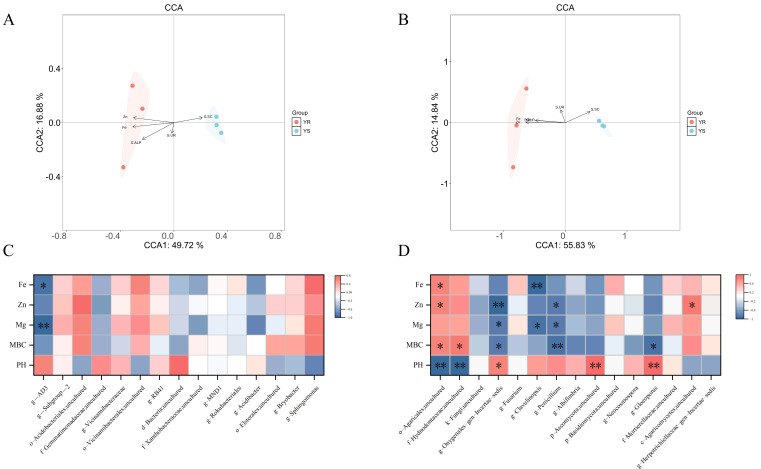
CCA and Spearman analysis of soil factors and microbial communities. (**A**) CCA analysis plot of soil physicochemical factors versus bacterial community structure. (**B**) CCA analysis plot of soil physicochemical factors versus fungal community structure. (**C**) Spearman correlation heatmap of bacteria with pH, MBC, Mg, Zn, and Fe. (**D**) Spearman correlation heatmap of fungi with pH, MBC, Mg, Zn, and Fe. * *p* < 0.05, ** *p* < 0.01.

CCA analysis of microbial OTU composition and fundamental properties across samples was conducted to describe the correlation between environmental factors and microbial community structure. In bacterial communities, two ordination axes explained 66.6% of the total variance (Figure 6A). In fungal communities, two axes explained 70.67% of the total variance (Figure 6B). From the results, we can speculate that zinc, iron, S-ALP, S-UR, and S-SC may be the key factors affecting bacterial and fungal communities. Notably, S-SC showed a significant positive correlation with microbial community structure in diseased areas, while Zn, Fe, S-ALP, and S-UR exhibited significant positive correlations with microbial community structure in healthy areas.

### 3.7. Correlation Analysis of Microbial Composition with Environmental Factors in Diseased YS and Healthy YR Rhizosphere Soil

At the genus level of the pepper rhizosphere soil microbial community, Spearman correlation analysis was conducted between the top 15 genera by relative abundance and five soil nutrient contents (pH, MBC, Mg, Zn, and Fe). The results are shown in Figure 6. In the bacterial community of pepper rhizosphere soil, only one bacterial genus showed significant correlations with soil nutrients: AD3 exhibited a highly significant negative correlation with Mg and a significant negative correlation with Fe (Figure 6C). In contrast, eight fungal genera in the pepper rhizosphere fungal community showed significant correlations with soil nutrients (Figure 6D). Agaricales, unculture, showed a highly significant negative correlation with pH and significant positive correlations with MBC, Zn, and Fe. Hydnodontaceae, uncultured, exhibited a highly significant negative correlation with pH and a significant positive correlation with MBC. Onygenales_gen_Incertae_sedis showed a positive correlation with pH and a significant negative correlation with MBC, Mg, and Zn. *Clavulinopsis* exhibited significant negative correlations with both Mg and Fe. *Penicillium* showed significant negative correlations with Mg and Zn and a highly significant negative correlation with MBC. Ascomycota, uncultured, and *Gloeoporus* both showed a highly significant positive correlation with pH, with the latter also exhibiting a significant negative correlation with MBC. Agaricomycetes, uncultured, showed a significant positive correlation with Zn.

## 4. Discussion

With the annual increase in pepper cultivation, pepper *Phytophthora* blight has emerged as a devastating soil-borne fungal disease, severely impacting both yield and quality [33]. While chemical control can achieve short-term disease prevention, prolonged use of chemical agents may enhance pathogen resistance and disrupt soil microbial structure and soil health [34]. Biological control methods, such as biocontrol agents, offer advantages including soil and environmental friendliness, non-toxicity to humans, animals, and plants, and reduced likelihood of pathogen resistance development [35]. Plant rhizosphere microorganisms typically play a crucial role in plant growth, development, and resistance to pathogen invasion. When pathogens infect plants, the plants can recruit antagonistic microorganisms from the soil to counteract the pathogens [36]. Many beneficial rhizosphere microorganisms are commercially available through cultivation methods to enhance crop nutrient uptake, improve stress tolerance, and defend against pathogen attacks [37]. Soil microbial diversity and specific species composition are closely linked to plant disease resistance [38]. Existing research indicates that disease-resistant soils often benefit from the protection of certain rhizosphere microorganisms. Conversely, soils conducive to disease development typically lack such protection, making diseased plants more vulnerable to pathogen attacks [39]. Understanding soil microbial diversity not only aids in elucidating potential mechanisms of plant disease occurrence but also facilitates the screening of beneficial rhizosphere microorganisms for plant disease control. Therefore, this study employs high-throughput sequencing technology to analyze the microbial community composition of soil in the rhizosphere of pepper plants infected with *Phytophthora capsici* and in healthy plants. This approach helps to elucidate the microbial community composition and differences between infected and healthy areas, identifies key species influencing the occurrence of pepper *Phytophthora* blight, and provides a theoretical foundation for applying microbiome regulation techniques to control pepper *Phytophthora* blight.

### 4.1. Potential Influence of Rhizosphere Soil Enzymes and Physicochemical Properties on Pepper Phytophthora Blight Occurrence

Soil enzymes serve as key drivers of metabolism within soil ecosystems [40]. Their activity is closely linked to soil physicochemical properties, indirectly reflecting how these properties regulate microbial activity [41]. S-ALP, S-SC, and S-UR all play vital roles in soil nutrient cycling. Among these, S-ALP [42] promotes phosphorus transformation, typically facilitating the hydrolysis of organophosphorus compounds for plant utilization. S-SC [43] participates in carbon conversion by hydrolysis of sucrose into soluble monosaccharides, and its activity reflects variations in soil organic matter as well as nitrogen and phosphorus nutrient contents. S-UR [44] catalyzes nitrogen transformation in soil, serving as an indicator of soil nitrogen nutrition; S-DHA [45] facilitates dehydrogenation reactions as an intermediate hydrogen carrier, playing a crucial role in organic matter decomposition. In this study, S-ALP, S-UR, and S-DHA activities in YR were higher than those in YS, while S-SC activity was lower than in YS, though no significant differences were observed (*p* > 0.05). Experiments by You Chunmei et al. demonstrated that in ginseng root rot disease, the activities of sucrase, dehydrogenase, phosphatase, and urease in the root zone soil of healthy plants were higher than those in diseased plants [46]. Research by Li Xueping et al. revealed that sucrase, urease, and alkaline phosphatase activities in the rhizosphere soil of barley decreased with the occurrence of root rot disease, whereas cellulase activity increased accordingly [47]. These findings are generally consistent with the results of this study. However, in this research, sucrase activity in the rhizosphere soil of diseased plants was higher than that of healthy plants. This may be attributed to the gradual decay and wilting of roots and stems in diseased plants, which provided substrates for sucrase and thereby enhanced its activity.

Soil physicochemical properties form a crucial foundation for sustainable agricultural production and ecosystem functions [48], playing a vital role in plant growth and the diversity of rhizosphere microbial communities [49]. Plants typically obtain nutrients through the mineralization and decomposition of organic forms of N, P, and K by soil microbial communities [12]. Existing research indicates that deficiencies in soil elements such as N, P, and K significantly increase the incidence of plant diseases [50]. Other readily available nutrients in soil include available iron, exchangeable calcium, and exchangeable magnesium [51]. The results of this study indicate that the EC value and Cu content of YR soil were lower than those of YS soil, while AN, AP, OM, Ca, and AK content were higher than those of YS soil, but no significant differences were observed (*p* > 0.05). pH value is an important basic physical and chemical property of soil, which directly affects the existence state, transformation process, and effectiveness of nutrients in soil. Therefore, it is of great significance to study the pH value of rhizosphere soil to understand the rhizosphere environment of plants [52]. In this study, the pH of YR soil was 7.967, significantly lower than the pH of YS soil (8.063; *p* < 0.05), representing a 1.191% reduction. Consistent with existing research, both excessively high and low soil pH levels can impair normal plant growth [53]. Research by Zhang, Guangyu et al. demonstrated that applying seaweed fertilizer and biochar significantly increased soil pH and nutrient content while reducing the disease index of bacterial wilt in tobacco [54]. Other studies indicate that disease severity diminishes as pH decreases [55]. Therefore, it can be inferred that soil and plant types influence the relationship between soil pH and plant disease occurrence. Under different soil and plant conditions, the optimal pH range and the impact of pH variations on disease differ. Currently, numerous domestic and international reports have documented the relationship between microbial biomass carbon and soil fertility, with microbial biomass carbon content regarded as a key indicator of soil fertility variation [56]. In this study, MBC, Mg, Zn, and Fe content in YR were significantly higher than those in YS (*p* < 0.05), with increases of 17.56%, 27.95%, 11.12%, and 30.30%, respectively. Research indicates that microorganisms can decompose organic matter in soil, releasing nutrients available for plant uptake and thereby enhancing the supply of available nutrients [57]. Consequently, the microbial biomass carbon and soil available nutrient content may exhibit strong mutual influence. Ma Yue et al. demonstrated that the occurrence of ginseng root rot is significantly related to soil physicochemical properties. Overall, the rhizosphere soil of healthy ginseng plants exhibited higher levels of alkaline-hydrolysable nitrogen, organic matter, available potassium, sucrase activity, and catalase activity compared to infected ginseng rhizosphere soil [58]. Research by Saiyaremu et al. found that uninfected soil samples exhibited superior physicochemical properties compared to infected samples, with significant seasonal variations. Environmental factors such as pH, OM, AP, and AK significantly influenced the fungal community structure in both uninfected and infected tree soils [59]. Soil physicochemical properties—including OM, AN, AP, AK, Ca, and Mg—were lower in the root zone soil of clubroot-infected Chinese cabbage plants compared to healthy plants [60], consistent with the findings of this study. In summary, preliminary evidence suggests a correlation between pepper *Phytophthora* blight occurrence and soil pH, MBC, Mg, Zn, and Fe content. Although this study indicates a close association between pepper *Phytophthora* blight incidence and soil physicochemical properties, further research is needed to determine whether the interaction is direct or indirect and whether the disease mechanism relates to the absorption and transformation of different soil nutrients.

### 4.2. Analysis of Soil Microbial Diversity and Dominant Species in YS and YR

Soil microbial communities constitute a vital component of the crop rhizosphere ecosystem, playing a crucial role in crop growth and development as well as pest and disease control. Existing research indicates that during soil-borne disease outbreaks, significant differences exist between healthy and diseased plants in terms of microbial diversity within the rhizosphere soil and the dynamic equilibrium between microbial communities [61,62]. Furthermore, soil fungal community diversity often correlates with the occurrence of soil-borne diseases [63]. Yu, Jinyang et al. demonstrated that compared to healthy soil, bacterial diversity decreased while fungal diversity and abundance increased in soil infected with garlic root rot in Pengzhou, Sichuan [64]. Tu, Zuxin et al. found that the fungal community diversity index in the rhizosphere soil of citrus plants infected with Huanglongbing was 23% higher than that in healthy plants [65]. Kang, Jie et al. observed that fungal diversity in the rhizosphere soil of two typical yam diseases was higher than in the control group, and certain fungal species may be associated with disease occurrence [66]. Generally, higher microbial community diversity indices indicate more complex community structures and greater system stability, reflecting enhanced resilience to environmental changes. In this study, the bacterial community diversity indices (Shannon and Simpson) of YR were higher than those in of YS. Among fungi, the microbial diversity of YR was significantly lower than that of YS (*p* < 0.05). These findings align with previous studies by Wu, Wenxian et al. [67] and Yang, Guangzhu et al. [68] on rhizosphere microbial communities of crops affected by soil-borne diseases. Therefore, we can preliminarily infer that the change in fungal community structure diversity may be a key factor affecting the quality of pepper rhizosphere soil. The infection of *Phytophthora* may lead to the increase in fungal diversity and the decrease in bacterial diversity in the rhizosphere soil of peppers. This change may further promote the occurrence of pepper *Phytophthora* blight by destroying the microecological balance.

To further investigate the relationship between pepper *Phytophthora* blight occurrence and rhizosphere soil microbial communities, we conducted species composition analysis of rhizosphere microbial communities from phylum to genus level. The study revealed that Acidobacteria was the dominant bacterial phylum in the soils of the study area. Acidobacteria constitutes a vital component of soil microbial communities, playing a significant role in soil material cycling and ecological environment construction. It possesses capabilities for degrading plant residues, participating in iron cycling, and supporting photosynthesis [69]. Not only can it improve soil aggregate formation, aeration, and water-retention properties, but its oligotrophic nature also enables competition with pathogens for resources, potentially inhibiting pathogen invasion. Proteobacteria exhibits exceptional metabolic diversity, encompassing nitrogen-fixing, nitrifying, and plant-promoting bacteria. Beneficial bacteria within this phylum, such as *Pseudomonas* [70] and *rhizobia* [71], can secrete antibiotics and induce resistance in plant systems to suppress pathogens. *Actinomycetes* enhances the utilization efficiency of soil nutrients and minerals [72]. *Burkholderia* typically exhibits fungicidal activity, directly inhibiting the proliferation of fungi [73]. *Rhodanobacteraceae* can act on plant–soil biochemical processes by synthesizing glycoside hydrolases to maintain the carbon cycle of soil ecology [74]. This study found that after pepper plants were infected with *Phytophthora capsici*, the relative abundance of Ascomycota fungi in the rhizosphere soil significantly increased, while the relative abundance of Basidiomycota significantly decreased. We hypothesize that healthy pepper roots may secrete substances inhibiting Ascomycota proliferation while promoting Basidiomycota growth. Upon infection, this balance between roots and rhizosphere microbes is disrupted, leading to Ascomycota proliferation. Chloroflexi exhibits diverse morphologies, extensive nutritional modes, and rich metabolic pathways. It is widely distributed across various habitats in the biosphere and participates in the biogeochemical cycling of elements such as carbon, nitrogen, and sulfur [75]. *Vicinamibacteraceae* belong to the Acidobacteria and are typically closely associated with soil nutrient decomposition, transformation, and plant nutrient uptake, thereby enhancing plant stress resistance [51]. In this study, both Chloroflexi and *Vicinamibacteraceae* were enriched in the rhizosphere soil of infected plants. This enrichment may result from the pepper plant activating its defense system upon pathogen invasion, releasing specific chemicals or root exudates to recruit beneficial rhizosphere microorganisms and enhance resistance against pathogens. Although GAL15 occupies an important ecological niche in infected areas, it is not a representative soil bacterium. Research on this bacterium is limited and its functions remain unclear, warranting further investigation. *Fusarium* is a common plant pathogen that typically synthesizes various toxic secondary metabolites to infect crops, causing diseases such as tobacco root rot [76] and watermelon wilt [77]. *Penicillium* often directly cause *penicillium* diseases in plants [78]. Cai Zeyan et al. identified numerous harmful fungal genera, including *Fusarium* and *Cladosporium*, in the susceptible pepper variety Fudi Jian—a finding consistent with the results presented herein [79]. In summary, at the genus classification level, plant pathogens such as *Fusarium* and *Penicillium* were significantly more abundant in YS. This indicates that plant pathogen damage is rarely singular; once soil disease-suppression capacity weakens or is lost, multiple disease threats emerge simultaneously.

### 4.3. Functional Prediction and Differential Analysis of Soil Microbial Communities in YS and YR

In this study, while focusing on the structure and composition of the soil microbial community, the microbial community function of the rhizosphere soil was predicted and analyzed based on PICRUSt2 and FUNGuild to explore the correlation between the potential function of microorganisms and the occurrence of pepper *Phytophthora* blight. Amino acid metabolism primarily involves the breakdown of amino acids into amines and carbon dioxide through processes such as deamination, transamination, and conjugated deamination, which are closely linked to plant nitrogen cycling [80]. In the YR group, the functional abundance of alanine, aspartate, and glutamate metabolism was significantly higher than in the YS group. Existing research indicates this metabolic pathway plays a pivotal role in plants, regulating not only respiration but also influencing nitrogen distribution and utilization efficiency. These processes are crucial for plant growth, development, and stress resistance [81]. Ansamycin belong to the macrolide class of antibiotics. Their backbone is constructed by type I polyketide synthase (PKS) using 3-amino-5-hydroxybenzoic acid (AHBA) as the starting unit. Members of this family generally exhibit significant antibacterial, antifungal, antitumor, and antiviral activities [82]. In contrast, vancomycin-type antibiotics are glycopeptide macromolecules that inhibit cell wall synthesis by binding to peptidoglycan precursors, serving as a critical therapeutic defense against severe infections caused by Gram-positive bacteria such as MRSA [83]. In this study, the relative abundance of biosynthesis of ansamycins and biosynthesis of vancomycin group antibiotics was higher in the YS group. We hypothesize that the occurrence of pepper *Phytophthora* blight may influence the secretion of related metabolites by rhizosphere microorganisms to defend against pathogen invasion, thereby enhancing plant disease resistance. Within fungal communities, the functional abundances of saprophytic and phytopathogenic groups were elevated in YS. This finding suggests that diseased pepper plants may favor the growth of saprophytic fungi and pathogens in the rhizosphere soil, disrupting soil microbial balance and thereby promoting disease progression.

### 4.4. Correlation Analysis Between Key Environmental Factors and Dominant Fungal Communities Reveals Mechanisms Underlying Pepper Phytophthora Blight Occurrence

Zn and Fe are essential micronutrients for plants, playing crucial roles in their growth and development. Zn is a component of multiple enzymes and is inextricably linked to the synthesis of plant auxins [84]. Existing research indicates that zinc-deficient plants are more susceptible to pathogen infection [85]. Research by Luo, Yifang et al. indicates that Zn inhibits the growth of *Phytophthora parasitica* var. *nicotianae* by suppressing the expression levels of the *csn4* and *csn7* genes and affecting antioxidant enzyme activity [86]. Zinc-containing agents typically exert direct effects on pathogens, activating plant defense mechanisms and reducing pathogen virulence, thereby effectively controlling plant diseases caused by fungal pathogens such as *Phytophthora* [87]. Fe, a constituent element of cytochromes and non-heme ferritins, participates in plant photosynthesis and respiration [88]. Fe typically plays a complex role in plant–pathogen interactions, as plants can utilize Fe to enhance localized oxidative stress as a defense against pathogens [89]. Other studies indicate that Fe can promote fungal growth in certain plant–fungal interactions [90]. Thus, increased Zn and Fe content not only promotes plant growth but also enhances plant immunity. While inhibiting pathogen growth, Zn and Fe also provide a more favorable growth environment for beneficial fungi, thereby promoting their proliferation. In this study, increased Zn and Fe levels in rhizosphere soil from healthy areas showed a positive correlation with the relative abundance of beneficial fungi such as Basidiomycetes. Conversely, after pepper plants were infected with *Phytophthora capsici*, the reduced Zn level in the soil favored the enrichment of pathogens like *Penicillium* species. This finding may have facilitated *Phytophthora* infection, leading to the occurrence of pepper *Phytophthora* blight.

**Figure 7:**
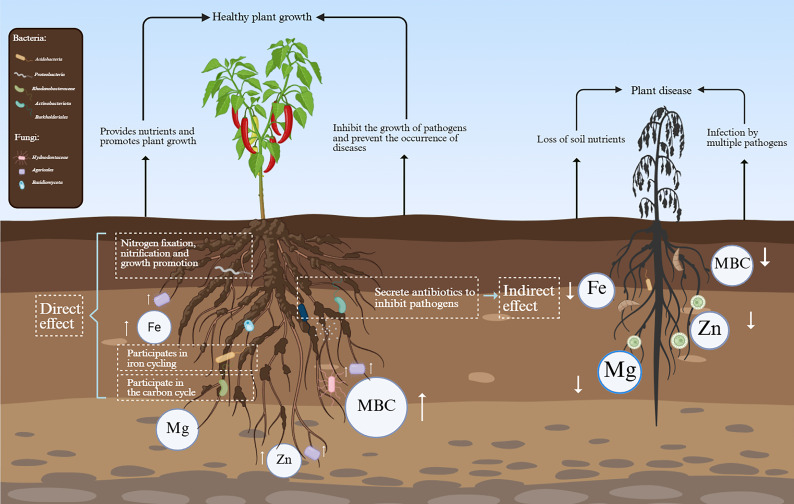
Schematic diagram of potential mechanisms by which pepper rhizosphere microorganisms and soil physicochemical factors influence pepper *Phytophthora* blight occurrence.

Combined with the above correlation analysis results, we plotted a schematic diagram of the interaction between soil physicochemical factors, enzyme activities, and rhizosphere microorganisms to reveal the possible mechanism of environmental and microbial factors in the occurrence of pepper *Phytophthora* blight (Figure 7). In summary, the contents of zinc and iron in rhizosphere soil may affect the occurrence of pepper blight by regulating the relative abundance of the beneficial bacteria Agaricales and the pathogenic bacteria *Penicillium*. It can be further inferred that significant changes in soil physical and chemical properties can affect the growth and metabolism of rhizosphere soil microorganisms and break the microecological balance. This will lead to an increase in the number of harmful microorganisms and pathogenic bacteria, and a decrease in the number of beneficial bacteria, thus seriously threatening plant health.

## 5. Conclusions

Following pepper *Phytophthora* blight infection, significant alterations occur in the physicochemical properties, microbial diversity, and composition of the pepper rhizosphere soil. Specifically, the rhizosphere soil pH of healthy pepper plants was significantly lower than that of diseased plants. When the pepper plant was infected by *Phytophthora capsici*, the contents of MBC, Fe, Zn, and Mg in the rhizosphere soil decreased significantly. Beneficial bacteria such as Burkholderiales and Rhodanobacteraceae under Proteobacteria were significantly enriched in the rhizosphere soil of healthy peppers. However, the relative abundance of pathogens such as *Penicillium* and *Fusarium* under Ascomycota was higher in the rhizosphere soil of diseased pepper plants. Based on CCA and Spearman correlation analysis, the Zn and Fe contents were significantly correlated with the rhizosphere microbial community structure. Therefore, we speculate that changes in the Zn and Fe contents may affect the growth metabolism and community balance of rhizosphere microorganisms by regulating the relative abundance of the beneficial bacteria Agaricales and the pathogenic bacteria *Penicillium* and may ultimately affect the occurrence of pepper *Phytophthora* blight. However, this hypothesis still needs to be further tested by relevant experiments. The above research results lay a foundation for the future research and application of biological control of pepper blight. However, how the dominant microorganisms interact with each other and how we can affect the mechanism of disease occurrence also needs to be further explored through subsequent experiments.

## Data Availability

All raw sequence data have been made available in the NCBI Sequence Read Archive (SRA) database under the bioproject accession number PRJNA1333628 and PRJNA1333613.

## Supplementary Materials

The following supporting information can be downloaded at https://www.mdpi.com/article/10.3390/microorganisms13122765/s1, Supplementary Figure S1: Distribution map of bacterial order-level taxonomic structure; Figure S2: Distribution map of bacterial family-level taxonomic structure; Figure S3: Distribution map of Fungal order-level taxonomic structure; Figure S4: Distribution map of Fungal family-level taxonomic structure.
