# Supplementary figures and images for: Study on the Influence Mechanism of the Rhizosphere Soil Microbial Community and Physicochemical Factors on the Occurrence of Pepper Phytophthora Blight
Source: Microorganisms. 2025 Dec 4;13(12):2765. doi: 10.3390/microorganisms13122765 (PMC12736286; doi:10.3390/microorganisms13122765)

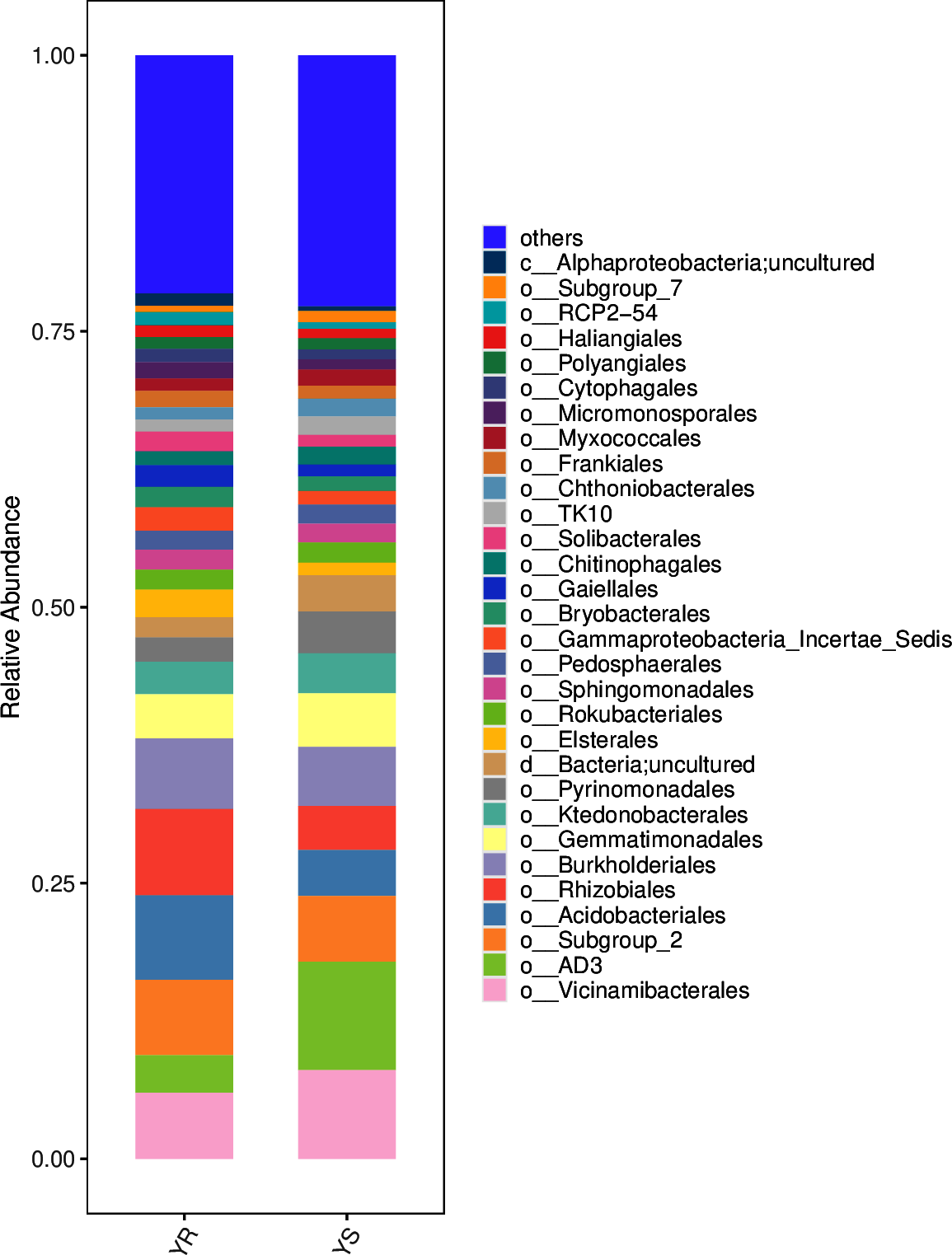

Supplement: Supplementary file 1 [file microorganisms-13-02765-s001.zip › S1 Distribution map of bacterial order-level taxonomic structure.png]

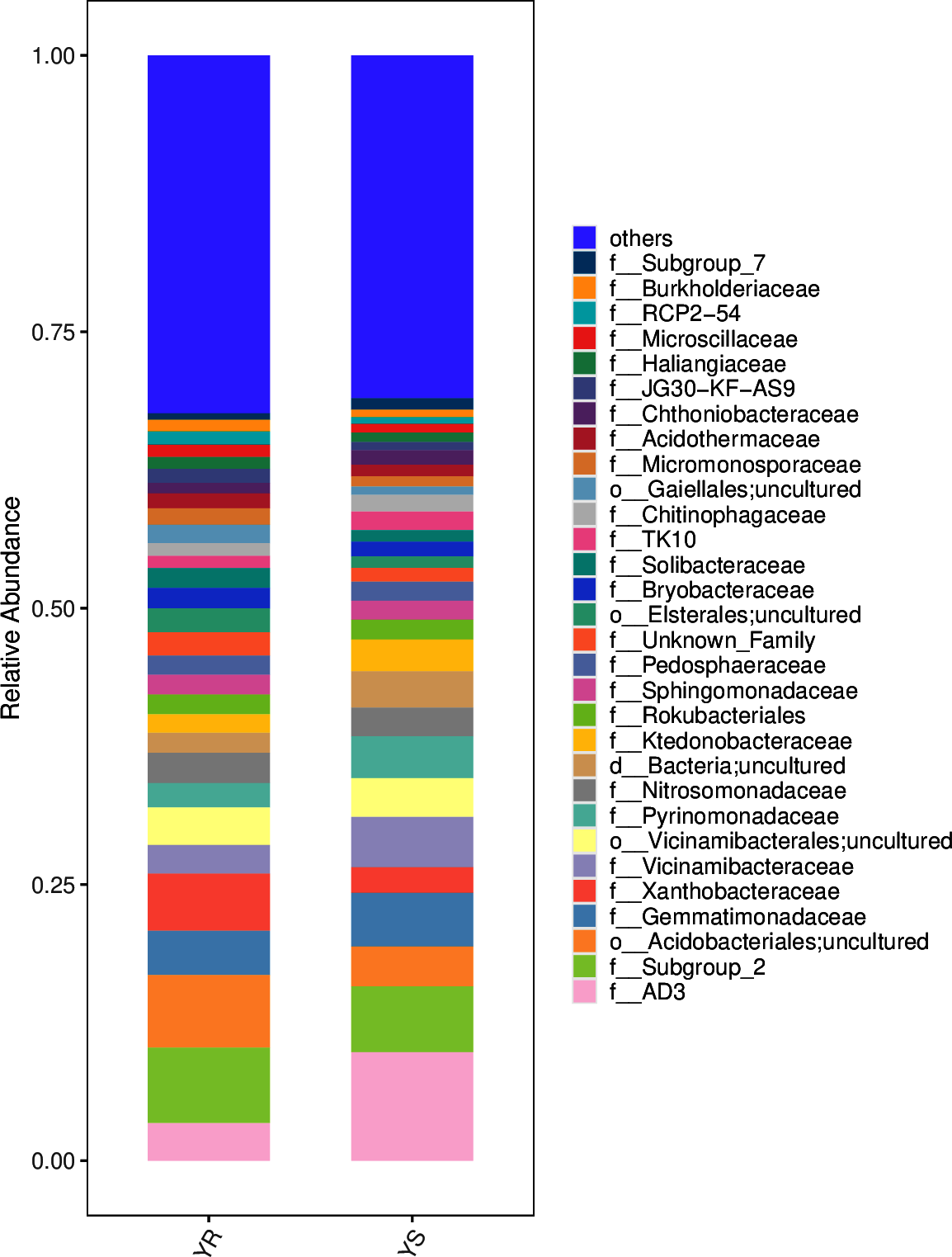

Supplement: Supplementary file 1 [file microorganisms-13-02765-s001.zip › S2 Distribution map of bacterial family-level taxonomic structure.png]

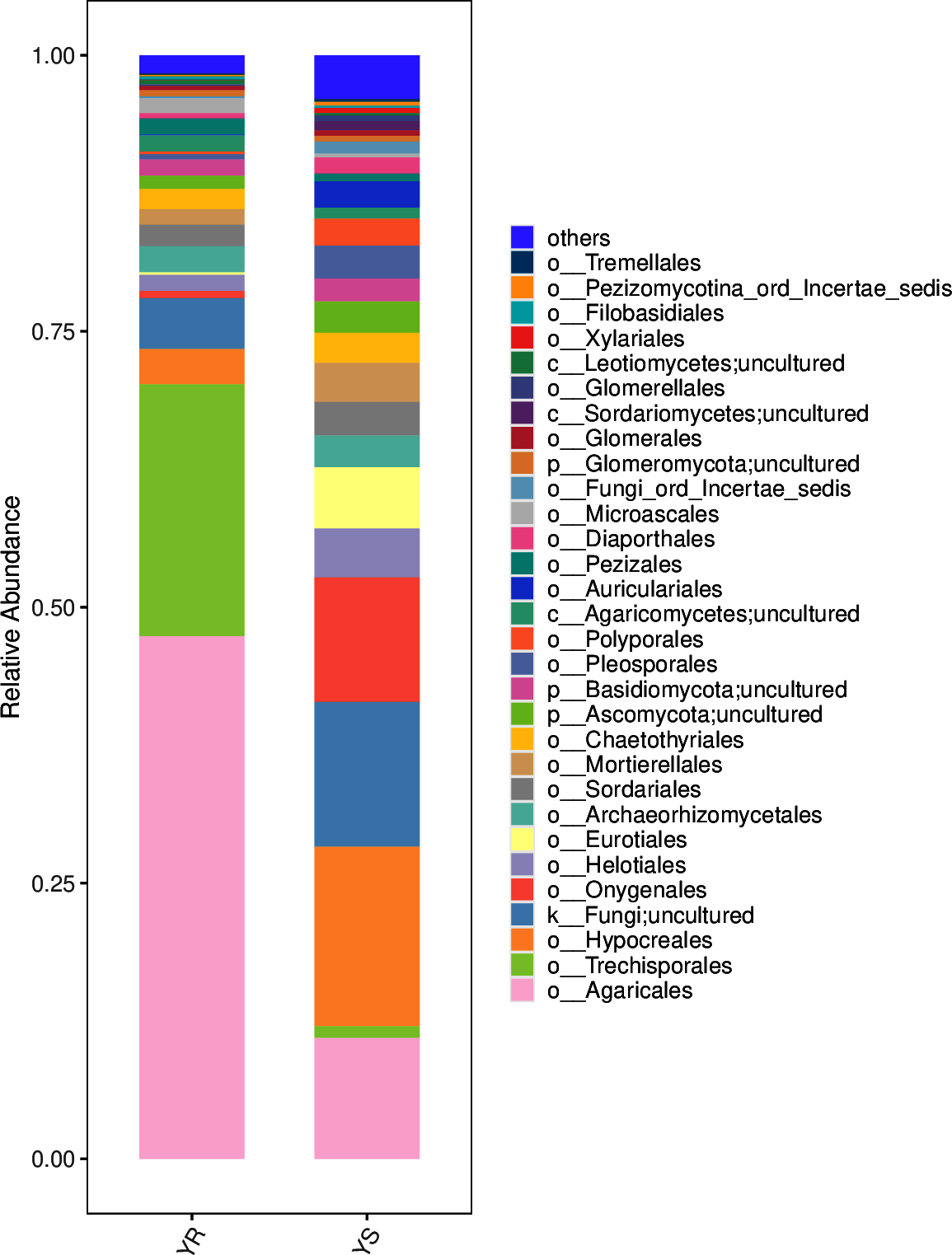

Supplement: Supplementary file 1 [file microorganisms-13-02765-s001.zip › S3 Distribution map of Fungal order-level taxonomic structure.png]

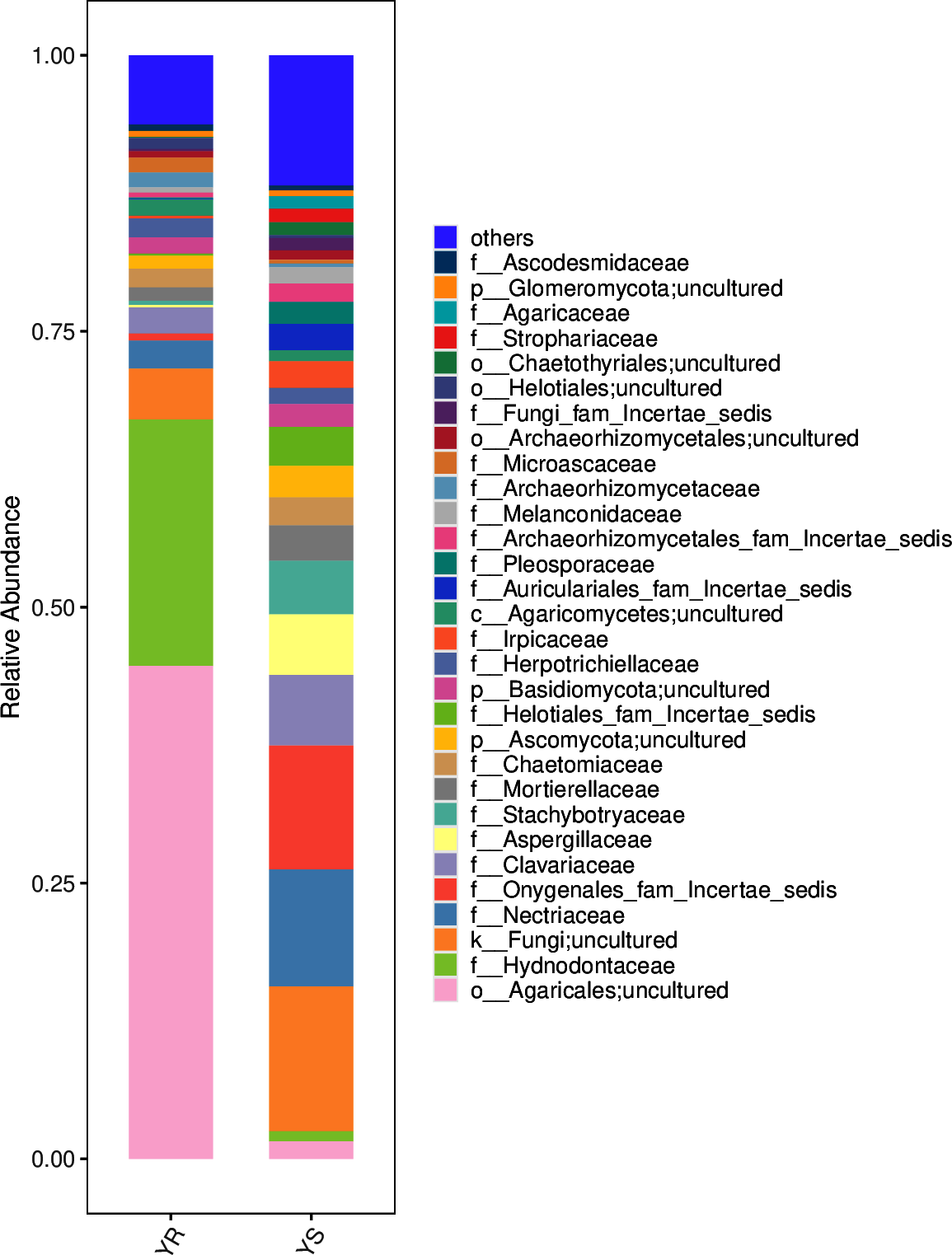

Supplement: Supplementary file 1 [file microorganisms-13-02765-s001.zip › S4 Distribution map of Fungal family-level taxonomic structure.png]
